# Specific retention of the protostome-specific *PsGEF *may parallel with the evolution of mushroom bodies in insect and lophotrochozoan brains

**DOI:** 10.1186/1741-7007-7-21

**Published:** 2009-05-07

**Authors:** Nozomu Higuchi, Keigo Kohno, Tatsuhiko Kadowaki

**Affiliations:** 1 Graduate School of Bioagricultural Sciences, Nagoya University, Chikusa, Nagoya 464-8601, Japan

## Abstract

**Background:**

Gene gain and subsequent retention or loss during evolution may be one of the underlying mechanisms involved in generating the diversity of metazoan nervous systems. However, the causal relationships acting therein have not been studied extensively.

**Results:**

We identified the gene *PsGEF *(protostome-specific GEF), which is present in all the sequenced genomes of insects and limpet but absent in those of sea anemones, deuterostomes, and nematodes. In *Drosophila melanogaster, PsGEF *encodes a short version of a protein with the C2 and PDZ domains, as well as a long version with the C2, PDZ, and RhoGEF domains through alternative splicing. Intriguingly, the exons encoding the RhoGEF domain are specifically deleted in the *Daphnia pulex *genome, suggesting that *Daphnia *PsGEF contains only the C2 and PDZ domains. Thus, the distribution of PsGEF containing the C2, PDZ, and RhoGEF domains among metazoans appears to coincide with the presence of mushroom bodies. Mushroom bodies are prominent neuropils involved in the processing of multiple sensory inputs as well as associative learning in the insect, platyhelminth, and annelid brains. In the adult *Drosophila *brain, *PsGEF *is expressed in mushroom bodies, antennal lobe, and optic lobe, where it is necessary for the correct axon branch formation of alpha/beta neurons in mushroom bodies. *PsGEF *genetically interacts with *Rac1 *but not other Rho family members, and the RhoGEF domain of PsGEF induces actin polymerization in the membrane, thus resulting in the membrane ruffling that is observed in cultured cells with activated forms of Rac.

**Conclusion:**

The specific acquisition of *PsGEF *by the last common ancestor of protostomes followed by its retention or loss in specific animal species during evolution demonstrates that there are some structural and/or functional features common between insect and lophotrochozoan nervous systems (for example, mushroom bodies), which are absent in all deuterostomes and cnidarians. *PsGEF *is therefore one of genes associated with the diversity of metazoan nervous systems.

## Background

A comparison of the genomes of five insects and five vertebrates revealed that there were approximately 1,000 genes present in all the insects but absent in the vertebrates. In contrast, there were approximately 5,000 genes present in all the vertebrates but absent in the insects [1]. The number of vertebrate-specific genes is five times larger than that of insect-specific genes, thus indicating that vertebrates have more complex gene pools than insects. Some of these genes have been acquired in order to support insect- and vertebrate-specific characteristics during evolution. However, if some of these vertebrate genes are shared with lophotrochozoans (the third large superphylum of Bilateria), this would imply that they were present in the last common ancestor of Bilateria (Urbilateria) and have been lost from insects during evolution [2,3]. A number of genes categorized to this group have already been reported and characterized [4]. Meanwhile, if some of these insect genes are shared with lophotrochozoans, this would suggest that they were specifically acquired by the last common ancestor of protostomes but not deuterostomes. Such genes have not been reported to date.

The origin and evolution of the metazoan central nervous system (CNS) have been intensively discussed. Large-scale expression analysis of neural genes in hemichordates has revealed that the mediolateral patterning genes (*Pax6, dbx, and msx*) and neural differentiation markers are expressed around the circumference of the embryo [5,6]. These results suggest that the centralization of a nervous system was acquired independently in deuterostomes and protostomes [7]. Meanwhile, several studies on the development of the CNS in *Drosophila melanogaster *and mouse have revealed common genetic patterning mechanisms in the formation of the insect and vertebrate brain. In both insects and vertebrates, the correct regionalization and neuronal identity of the anterior brain region is regulated by the cephalic gap genes *otd/Otx *and *ems/Emx*, whereas patterning of the posterior brain involves members of the *Hox *genes [8]. A recent study on gene expression patterns in the brain of developing annelids (*Platynereis*) has demonstrated that the patterning mechanism of the CNS is well conserved among chordates and annelids [9]. These studies strongly indicate that Urbilateria already had an anatomically complex CNS. Furthermore, cross-species comparisons of genome sequences and expressed sequence tag data sets have demonstrated the presence of a common ancestral CNS at the molecular level [3,10]. These results suggest that Urbilateria and the last common ancestor of protostomes were genetically complex, and have complex nervous systems [4].

Mushroom bodies (MBs) are lobed neuropils that comprise long and approximately parallel axons originating from clusters of minute basophilic cells located dorsally in the most anterior neuromere of the CNS. Structures with these morphological properties are found in many marine annelids (lophotrochozoa) and almost all arthropods (ecdysozoa) except crustaceans [11]. MBs are higher multisensory centers (for example, olfaction and vision) of the insect brain and are implicated in olfactory and other forms of associative learning [12]. There are two possibilities regarding the presence of MBs in different animal lineages (arthropod groups except crustacea and lophotrochozoa). One possibility is that the ancient MB-like structure was present in the CNS of the last common ancestor of all protostomes, and then some species have evolved the present MBs but the others have lost it secondarily during evolution. Another possibility is that MBs have independently evolved several times in different animal lineages by convergent evolution. It was reported that several genes encoding transcription factors, *eyeless (ey), twin of eyeless (toy)*, and *dachsund (dac)*, are necessary for the development of the *Drosophila *MB [13-15]. Their homologs are present in various metazoan genomes, and *Pax6 *(the vertebrate homolog of *ey*), for example, also has essential roles for neural development [16]. To prove the single origin of MBs, it will be necessary to demonstrate that the expression domains of the above genes are conserved in the arthropod and lophotrochozoan CNS during development.

Here, we report a novel gene, namely *PsGEF *(GEF, guanine nucleotide exchange factor), which is present in insect and *Lottia *(lophotrochozoa) genomes but absent in *Nematostella *(cnidaria), deuterostome, and nematode genomes. It is likely that *PsGEF *was specifically acquired by the last common ancestor of protostomes, and then lost in some species, for example nematodes. Intriguingly, the presence of PsGEF containing the C2, PDZ, and RhoGEF domains appears to coincide with the presence of MBs. Further, in *Drosophila*, PsGEF functions as a GEF for Rac and is essential for axon development in MBs. These results suggest that gain, retention, and loss of *PsGEF *are associated with some structural and/or functional features common between insect and lophotrochozoan nervous systems, which are absent in all deuterostomes and cnidarians. Thus, *PsGEF *is one of candidate genes associated with the diversity of metazoan nervous systems.

## Results

### Identification of PsGEF gene uniquely shared between insects and limpets

A large-scale comparison of the genomes of five insects (*D. melanogaster, Anopheles gambiae*, *Aedes aegypti*, *Apis mellifera*, and *Tribolium castaneum*) and five vertebrates (*Homo sapiens*, *Mus musculus*, *Monodelphis domestica*, *Gallus gallus*, and *Tetraodon nigroviridis*) revealed that there were approximately 1,000 insect-specific orthologous genes [1]. We searched among these genes for those that are highly expressed in the *Drosophila *CNS by screening transgenic lines in which *GAL4 *was inserted in the promoter regions of candidate genes, in order to understand the genetic basis for the development and functions specific for the insect nervous system. From the screening, we found one gene, the *PsGEF *gene (*CG14045*).

**Figure 1:**
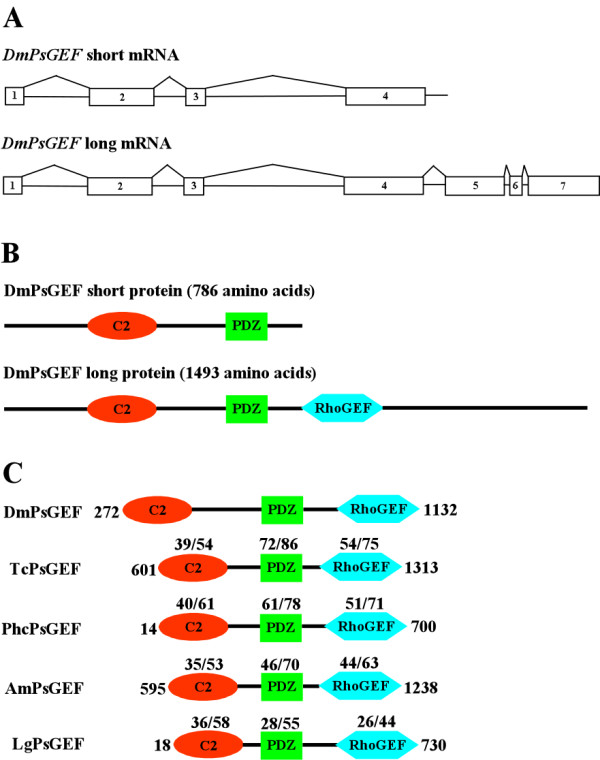
**Structure of two different *Drosophila PsGEF (DmPsGEF) *mRNAs and proteins and comparison of C2, PDZ, and RhoGEF domains of 5 PsGEFs from different species**. **(A) **Exon-intron organization and splicing pattern of *DmPsGEF*. The rectangles with numbers represent the exons, and the straight lines between the exons represent the introns. Polyadenylation at the 3' ends of exon 4 and exon 7 results in the synthesis of short and long *DmPsGEF *mRNAs, respectively. **(B) **Domain organizations of DmPsGEF proteins. DmPsGEF short protein with 786 amino acids contains the C2 (red oval) and PDZ (green rectangle) domains, and DmPsGEF long protein with 1493 amino acids also contains the RhoGEF domain in addition (blue hexagon). **(C) **Comparison of C2, PDZ, and RhoGEF domains of DmPsGEF, TcPsGEF (*Tribolium *PsGEF), PhcPsGEF (*Pediculus *PsGEF), AmPsGEF (*Apis *PsGEF), and LgPsGEF (*Lottia *PsGEF). The amino acid sequences containing the C2, PDZ, and RhoGEF domains of TcPsGEF (amino acid 601 to 1313), PhcPsGEF (amino acid 14 to 700), AmPsGEF (amino acid 595–1238), and LgPsGEF (amino acid 18 to 730) proteins are compared with those of DmPsGEF (amino acid 272 to 1132). The numbers above C2, PDZ, and RhoGEF domains of TcPsGEF, PhcPsGEF, AmPsGEF, and LgPsGEF proteins represent % identity/% similarity of their amino acid sequences to those of DmPsGEF. See also Additional file 1 for the full-length amino acid sequences of five PsGEF proteins and Additional file 2 for alignment of above amino acid sequences.

*Drosophila PsGEF (DmPsGEF) *encodes a protein with the C2, PDZ, and RhoGEF domains (Figure 1B). The C2 domain is a Ca^2+^-dependent membrane-targeting module found in many proteins involved in signal transduction or membrane trafficking [17]. It is thought to be involved in Ca^2+^-dependent phospholipid binding and in membrane-targeting processes [18]. The PDZ domain mediates binding with other proteins and is found in many signaling proteins frequently associated with the plasma membrane [19]. Moreover, it is often associated with scaffolding proteins important for synaptic development [20]. The RhoGEF domain activates Rho family GTPases, namely, Rho, Rac, and Cdc42, through release of bound guanosine diphosphate and subsequent binding of guanosine triphosphate [21]. Thus, RhoGEF activity of PsGEF appears to require an increase in the intracellular Ca^2+ ^level as well as association of PsGEF with other proteins. As shown in Figures 1A and 1B, two different *DmPsGEF *transcripts (short and long mRNAs consisting of four and seven exons, respectively) are found. The short mRNA encodes a 786-amino acid protein containing the C2 and PDZ domains, and the long mRNA encodes a 1493-amino acid protein containing the C2, PDZ, and RhoGEF domains. These two types of *DmPsGEF *mRNAs appear to be synthesized by alternative polyadenylation; polyadenylation at the 3' end of exons 4 and 7 results in the synthesis of short and long *DmPsGEF *mRNAs, respectively (Figure 1A). The short PsGEF with C2 and PDZ domains exhibits significant similarity to the vertebrate RGS3, which also contains C2 and PDZ domains [22]. However, PsGEF lacks amino acid sequences necessary for constituting the regulator of G-protein signaling (RGS) domain together with the C2 and PDZ domains, thus suggesting that the short PsGEF does not function as an RGS.

*DmPsGEF *orthologs are also present in the genomes of *Nasonia vitripenis *(parasitic wasp), *Pediculus humanus corporis *(human body louse), and *Acyrthosiphon pisum *(pea aphid). The proteins encoded by these contain C2, PDZ, and RhoGEF domains, similar to the long version of the DmPsGEF. *PsGEF *was therefore present in the common ancestor of holometabolous and hemimetabolous insects. Further, we searched for insect *PsGEF *orthologs in the genomes of *Nematostella vectensis *(sea anemone, cnidaria), *Strongylocentrotus purpuratus *(sea urchin), *Ciona intestinalis *(sea squirt), *Lottia gigantea *(limpet, lophotrochozoa), and *Caenorhabditis elegans*. We have found that only *Lottia *contains an insect *PsGEF *ortholog coding for a protein containing the C2, PDZ, and RhoGEF domains. As PsGEF has a complex domain organization, it may be difficult to identify the orthologs if they contain very large introns in some cases. *PsGEF *is present in insects (ecdysozoa) and limpet (lophotrochozoa) but not sea anemone or deuterostomes, suggesting that *PsGEF *was specifically acquired by the last common ancestor of protostomes. *Drosophila *(DmPsGEF), *Tribolium *(TcPsGEF), *Pediculus *(PhcPsGEF), *Apis *(AmPsGEF), and *Lottia *(LgPsGEF) PsGEFs (Additional file 1) share the C2, PDZ, and RhoGEF domains as shown in Figure 1C. The alignment of amino acid sequences containing the above functional domains of five PsGEF proteins demonstrates that they show significant similarity only in the C2, PDZ, and RhoGEF domains (Additional file 2). This was also the case when the full-length amino acid sequences were analyzed (data not shown).

**Figure 2:**
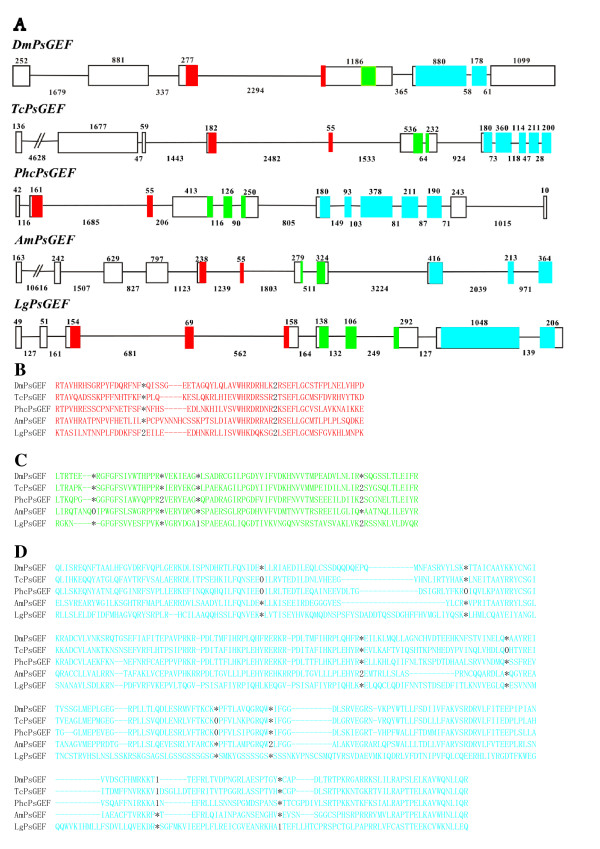
**Exon-intron organizations as well as intron positions of five *PsGEFs *from different species**. **(A) **Exon-intron organizations of *DmPsGEF, TcPsGEF, PhcPsGEF, AmPsGEF*, and *LgPsGEF*. The exon and intron are represented by rectangle and straight line, respectively. Exon 1 is on the left. The numbers above exons and below introns indicate their sizes, and thus their scales are different among five *PsGEFs*. The exon sequences encoding the C2, PDZ, and RhoGEF domains are highlighted by red, green, and blue, respectively. The initiation codon is present in the exon 1 except *DmPsGEF *in which it is located in the exon 2. **(B to D) **Intron positions within the C2 (B), PDZ (C), and RhoGEF (D) domain-coding regions of *DmPsGEF, TcPsGEF, PhcPsGEF, AmPsGEF*, and *LgPsGEF *are indicated by digits corresponding to the phase of the intron relative to the surrounding codons (phase 0, 1, and 2 introns fall before the first, second, and third bases of a codon, respectively). Asterisk indicates the absence of intron. Some intron positions are conserved at the exact homologous positions and phases between five *PsGEFs*.

The exon-intron organizations of the above five *PsGEF *genes are shown in Figure 2A. Insect and limpet *PsGEFs *contain one and two introns in the C2 domain-coding regions, respectively. Intriguingly, the position of one out of two introns is conserved at the same phase in five species (Figure 2B). Nevertheless, the size of this particular intron is varied, ranging from 562 to 2,482 base pairs. *DmPsGEF *has no intron, *TcPsGEF *and *AmPsGEF *have one intron, and *PhcPsGEF *and *LgPsGEF *contain two introns in the PDZ domain-coding regions (Figure 2A). *TcPsGEF, PhcPsGEF*, and *LgPsGEF *share one intron position as well as phases among them (Figure 2C). *DmPsGEF *and *LgPsGEF *contain one intron, *AmPsGEF *has two introns, and *TcPsGEF *and *PhcPsGEF *contain four introns in the RhoGEF domain-coding regions (Figure 2A). Among them, one intron position is conserved at the same phase in *DmPsGEF, TcPsGEF*, and *PhcPsGEF*. In addition, the positions and phases of two introns are conserved in *TcPsGEF *and *PhcPsGEF*. Thus, *TcPsGEF *and *PhcPsGEF *share the same positions and phases of three out of four introns (Figure 2D). These results demonstrate that some but not all intron positions are conserved at the same phase in the C2, PDZ, and RhoGEF domain-coding regions of five *PsGEFs *from different species.

### Daphnia PsGEF lacks RhoGEF domain

**Figure 3:**
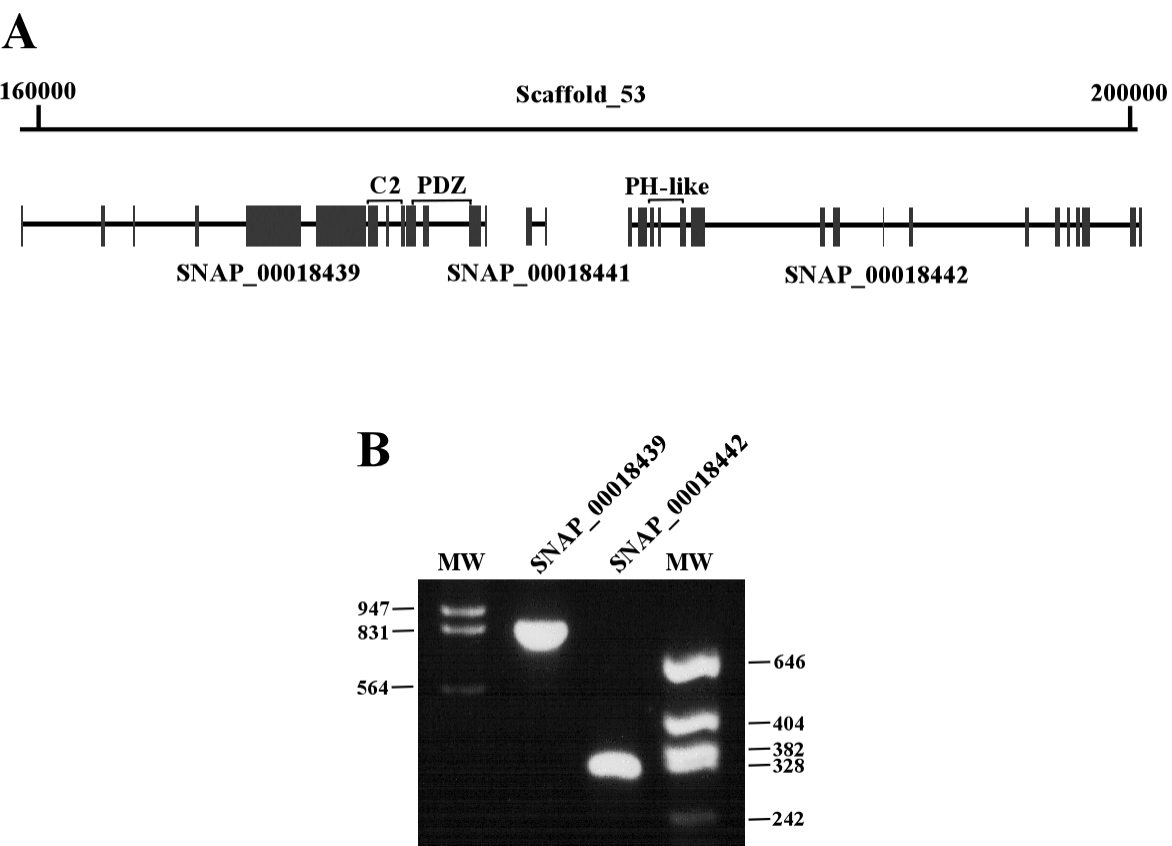
***Daphnia pulex *genomic DNA region containing *PsGEF *and mRNA expression of *Daphnia PsGEF *and neighboring genes**. **(A) ***Daphnia PsGEF *is located in scaffold 53 of the genome assembly. The predicted transcript *SNAP_00018439  *encodes PsGEF containing the C2 and PDZ domains. The neighboring transcripts *SNAP_00018441 *and *SNAP_00018442 *encode a novel short protein and a protein with a PH-like domain, respectively. The predicted exons are indicated by the solid rectangles. The exons encoding the C2, PDZ, and PH-like domains are indicated by brackets. The exons encoding the RhoGEF domain are not present in this scaffold. **(B) **The expression of SNAP_00018439and *SNAP_00018442 *mRNAs in *Daphnia *is confirmed by using RT-PCR. However, *SNAP_00018441 *mRNA can not be detected by this analysis. The numbers at both sides of the panel indicate the sizes of bands in molecular weight markers (MW).

We next analyzed the *PsGEF *ortholog in *Daphnia pulex*, which belongs to crustacea, one of major arthropod groups. The *PsGEF *ortholog is present in scaffold 53 of the *Daphnia pulex *genome assembly; however, the exons encoding the RhoGEF domain are apparently missing (Figure 3A). According to the JGI database [23], three predicted genes (*SNAP_00018439, SNAP_00018441*, and *SNAP_00018442*) are present in this genomic region. *A SNAP_00018439 *mRNA was detected by using reverse transcriptase-polymerase chain reaction (RT-PCR) and appears to encode the PsGEF protein containing only the C2 and PDZ domains. *SNAP_00018442*, but not *SNAP_00018441 *mRNA was detected by RT-PCR, and it encodes a novel protein with a PH-like domain (Figure 3B).

The above results indicate that PsGEF with the C2, PDZ, and RhoGEF domains is specifically present in lophotrochozoan and insect genomes but not in crustacean genomes. This distribution among metazoans accurately coincides with the presence of MBs, prominent neuropils involved in processing multiple sensory inputs as well as associative learning in the insect, platyhelminth, and annelid brains [11]. We therefore tested whether PsGEF plays a role in the development or functions of MB in *Drosophila*.

### Region-specific expression of DmPsGEF in the embryonic and adult central nervous system

**Figure 4:**
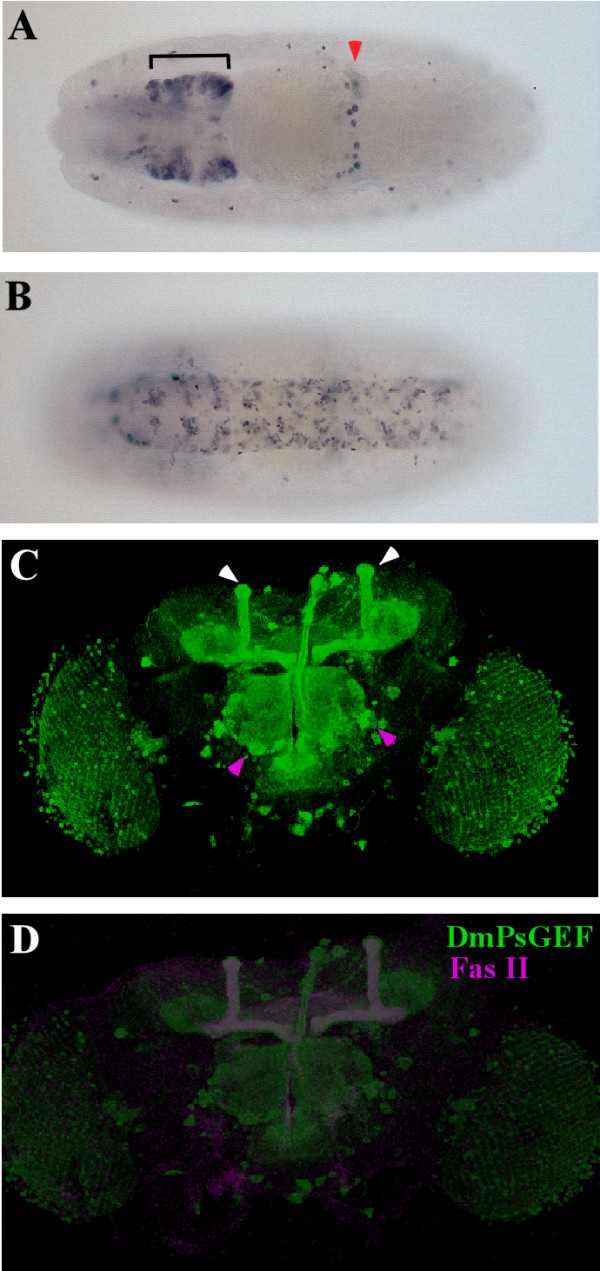
**Expression patterns of *DmPsGEF *in *Drosophila***. **(A to D) **The expression of *DmPsGEF *is examined in progenies carrying *NP5114 *and *UAS-mCD8::GFP. DmPsGEF *is expressed in the subsets of cells in the brain (A, bracket) and the ventral nerve cord (B) as well as cells at the midgut fusion point (A, red arrowhead) of the stage-15 embryos. The dorsal and ventral views are shown in (A) and (B), respectively. The anterior view is on the left. *DmPsGEF *is expressed in the optic lobes, antennal lobes, discrete neurons surrounding the antennal lobes (C, magenta arrowheads), and mushroom bodies (C, white arrowheads) in the adult brain. *DmPsGEF *(indicated in green) is primarily expressed in Fas II-positive alpha/beta neurons (shown by magenta) in mushroom bodies (MBs) (D).

We identified several *GAL4 *enhancer trap lines (*NP5114, NP0264, NP1088, NP7169, NP3316, NP7265, NP3612*, and *NP3237*) located in the promoter region of *DmPsGEF*. These *GAL4 *lines were crossed with *UAS-mCD8-GFP *lines to detect the expression of *DmPsGEF *in embryos and adults, and all of them exhibited the same expression patterns. *DmPsGEF *is expressed in the subsets of cells in the brain and ventral nerve cord as well as cells at the midgut fusion point in the stage-15 embryos (Figures 4A and 4B). *DmPsGEF *is highly expressed in MBs, the antennal lobe, and the optic lobe of the adult brain. In addition, there are several large discrete *DmPsGEF-*positive cells surrounding the antennal lobe (Figure 4C). The presence of *DmPsGEF-*positive lobes of MBs, as observed by staining for Fasciclin II (Fas II), suggested that *DmPsGEF *is expressed in the alpha/beta neurons of MBs (Figure 4D). *AmPsGEF *mRNA is also highly expressed in the adult honey bee brain (data not shown).

### DmPsGEF is necessary for the axon development of mushroom bodies

**Figure 5:**
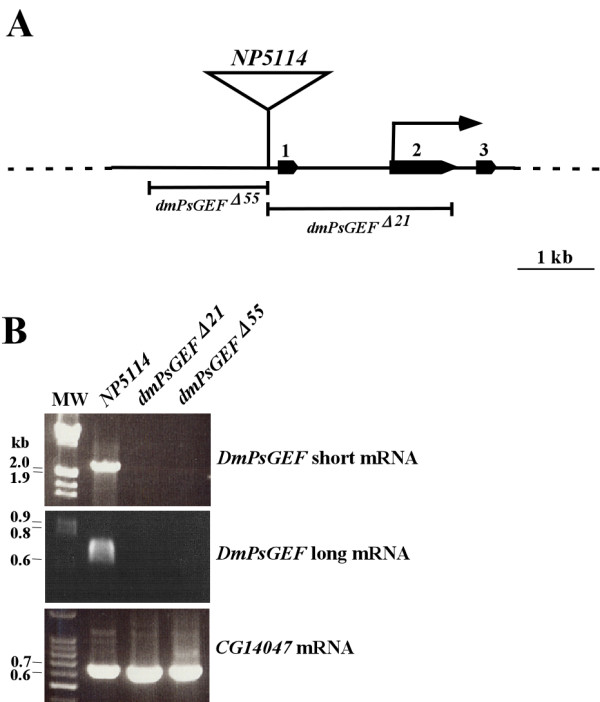
**Generation of *DmPsGEF *deletion mutants and *DmPsGEF *mRNA expression analysis in the mutants**. **(A) ***NP5114 *is located at 5' upstream of exon 1 of *DmPsGEF*, and translation is initiated at exon 2 (arrow). Exons 1 to 3 are indicated by solid pentagons. Two *DmPsGEF *deletion mutants (*dmPsGEF*^Δ*55 *^and *dmPsGEF*^Δ*21*^) were generated by imprecise excision of *NP5114*, and the deleted genomic region in each mutant is also shown. The scale bar indicates 1 kb. **(B) **The expression of short and long *DmPsGEF *mRNAs as well as *CG14047 *mRNA in *NP5114*, *dmPsGEF*^Δ*21*^, and *dmPsGEF*^Δ*55 *^embryos was examined by reverse transcriptase-polymerase chain reaction. Both short and long *DmPsGEF *mRNAs are present in the parent *NP5114 *but not in *dmPsGEF*^Δ*21 *^and *dmPsGEF*^Δ*55 *^embryos, while *CG14047 *mRNA is present in all genotypes of embryos. The numbers at left side of the panels indicate the sizes of bands (in kb) in molecular weight markers (MW).

To understand the functions of DmPsGEF, we generated *DmPsGEF *loss-of-function mutants by imprecise excision of *NP5114*. Two deletion mutants were recovered. The deletion mutants included *dmPsGEF*^Δ*55*^, in which 1.5 kb genomic DNA containing the promoter region is deleted, and *dmPsGEF*^Δ*21*^, in which 2.7 kb genomic DNA containing exons 1 and 2 is deleted (Figure 5A). Both *dmPsGEF*^Δ*55 *^and *dmPsGEF*^Δ*21 *^are viable and fertile with no morphological defects. The expression of *DmPsGEF *mRNA was examined in *NP5114, dmPsGEF*^Δ*55*^, and *dmPsGEF*^Δ*21 *^embryos by RT-PCR. Both short and long mRNAs are expressed in the *NP5114 *embryos; however, they are absent in *dmPsGEF*^Δ*55 *^and *dmPsGEF*^Δ*21 *^embryos. The adjacent *CG14047 *mRNA is equally expressed in all embryos (Figure 5B). These results suggest that both *dmPsGEF*^Δ*55 *^and *dmPsGEF*^Δ*21 *^are *DmPsGEF*-null alleles.

**Figure 6:**
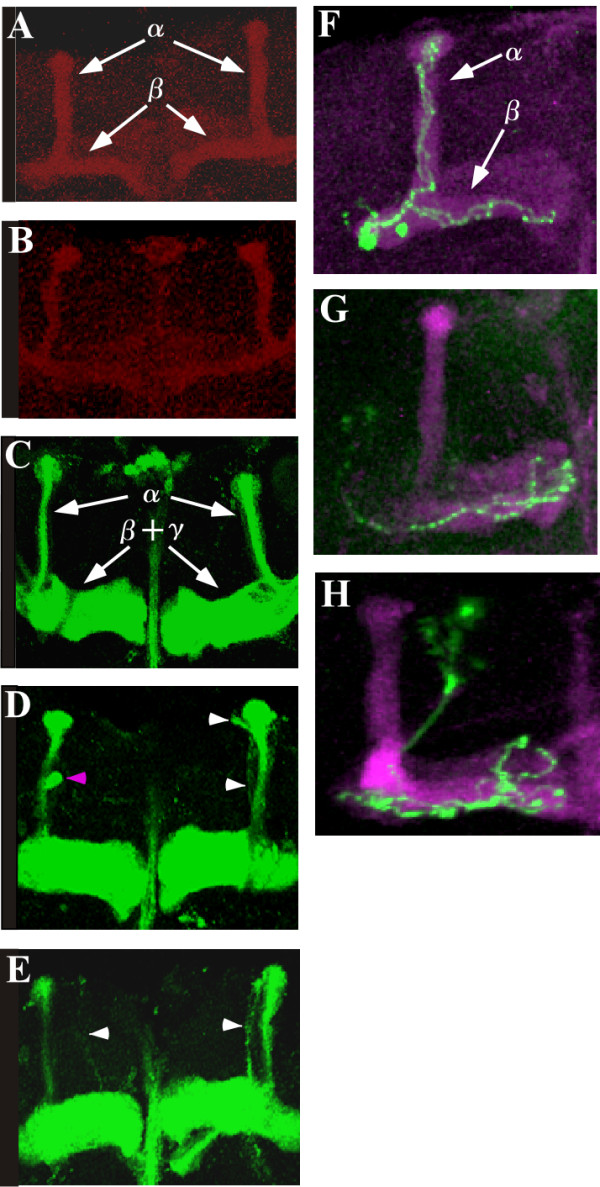
**Morphology of alpha/beta lobes and alpha/beta neurons in mushroom bodies of the wild-type and the *dmPsGEF*^Δ*21 *^mutant**. The alpha lobes detected by Fas II staining are thinner in the *dmPsGEF*^Δ*21 *^mutants **(B) **than in the wild-type **(A)**. The alpha and beta lobes are indicated by arrows in (A). The alpha lobes of late-born alpha/beta neurons are visualized by *201Y-Gal4 *and *UAS-mCD8::GFP *in the wild-type **(C) **and *dmPsGEF*^Δ*21 *^mutants **(D and E)**. The alpha lobes are thinner in *dmPsGEF*^Δ*21 *^mutant than in the wild-type, as observed above. In addition, the alpha lobes are often short (magenta arrowhead in D), and their positions along the beta lobes vary (white arrowheads in D and E). The alpha as well as the beta and gamma lobes are indicated by arrows in (C). Findings of the repressible cell marker analysis reveal that the axons of wild-type alpha/beta neurons (green) bifurcated toward the alpha/beta lobes (magenta) **(F)**. However, the axons of *dmPsGEF*^Δ*21 *^alpha/beta neurons (green) often fail to grow toward the alpha lobes **(G and H)**. The alpha and beta lobes are indicated by arrows in (F).

Since *DmPsGEF *is highly expressed in the alpha/beta neurons of MBs (Figures 4C and 4D), we analyzed the morphology of the alpha/beta lobes in *dmPsGEF*^Δ*21 *^hemizygous males by immunostaining of Fas II. The Fas II-positive alpha/beta lobes are thinner in *dmPsGEF*^Δ*21 *^than in wild-type males (Figures 6A and 6B). Furthermore, the alpha lobes of late-born alpha/beta neurons visualized by *201Y-GAL4 *and *UAS-mCD8-GFP *are thinner in *dmPsGEF*^Δ*21 *^because the alpha lobes are often short, and their positions along the beta lobes vary (Figures 6C to 6E). The same results were obtained for *dmPsGEF*^Δ*55 *^(data not shown). We also analyzed the morphology of the alpha'/beta' lobes in *dmPsGEF*^Δ*21 *^and wild-type males by anti-Trio antibody staining [24]. No significant difference was observed (data not shown).

For a more accurate analysis of the role of DmPsGEF in the development of MBs, the morphology of alpha/beta neurons at a single-cell level was examined by using the mosaic analysis with a repressible cell marker (MARCM) system [25]. The wild-type alpha/beta neurons bifurcate their axons into the alpha and beta lobes (Figure 6F); however, 34% of late-born *dmPsGEF*^Δ*21 *^alpha/beta neurons (*n *= 35) have branching defects, and the axons toward the alpha lobe are missing (Figures 6G and 6H). These results demonstrate that DmPsGEF is necessary for the correct axonal development of alpha/beta neurons in MBs.

### DmPsGEF genetically interacts with Rac1 but not other Rho family members for the axon development in mushroom bodies

**Figure 7:**
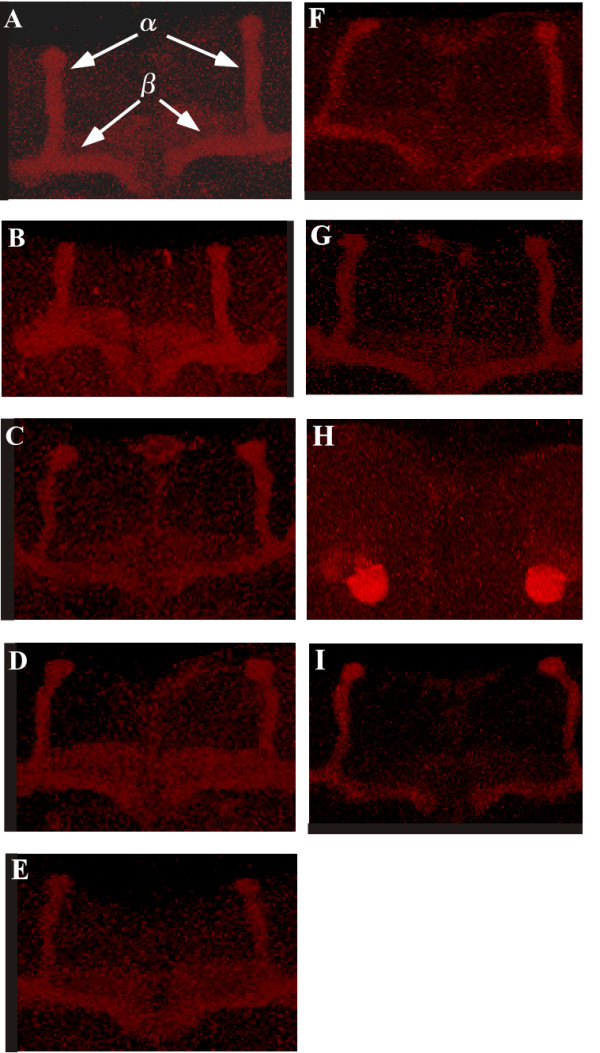
**Genetic interaction of *DmPsGEF *with *Rho *family members**. The morphology of the alpha/beta lobes of mushroom bodies in wild-type **(A)**, *rac1*^*J11*^*/+ ***(B)**, and *dmPsGEF*^Δ*21 *^hemizygous males **(C) **as well as under *rho1*^*E3.10 *^**(D)**, *rac2*^Δ ^**(E)**, *mtl*^Δ ^**(F)**, *rac1*^*J11 *^**(H)**, and *pak*^6 ^**(I) **heterozygous background is shown. To determine the interactions with *Cdc42 ***(G)**, *dmPsGEF*^Δ*21 *^homozygous females with *cdc42*^4 ^heterozygous background were analyzed. The alpha and beta lobes are indicated by arrows in (A). The reduction of *Rac1 *gene dosage dramatically influences the phenotypes; a pair of alpha/beta lobes is missing in 90% of the examined animals (H).

As PsGEF is expected to function as a RhoGEF, we analyzed the genetic interactions of *DmPsGEF *with *Rho *family members. We thus examined the morphology of the alpha/beta lobes in the MBs of *dmPsGEF*^Δ*21 *^hemizygous males in a *rho1*^*E3.10*^, *rac1*^*J11*^, *rac2*^Δ^, and *mtl*^Δ ^heterozygous background. For the interaction with *Cdc42, dmPsGEF*^Δ*21 *^homozygous females with a *cdc42*^*4 *^heterozygous background were analyzed. It was observed that reducing the gene dosages of *Cdc42, Rho1, Rac2*, and *Mtl *does not affect the phenotypes of the alpha/beta lobes as observed with *dmPsGEF*^Δ*21 *^(Figures 7A and 7C to 7G). However, reduction in the *Rac1 *gene dosage dramatically influences the phenotypes: a pair of alpha/beta lobes was missing in 90% of the animals examined (Figure 7H). It was observed that 65% of *rac1*^*J11*^*/+ *heterozygotes have normal alpha/beta lobes (Figure 7B), and 35% have the branching defects as previously reported [26]. These results demonstrate that *DmPsGEF *genetically interacts with *Rac1*. However, *DmPsGEF *does not exhibit genetic interaction with *Pak*, which is a downstream effector of Rac1 (Figure 7I). This suggests that DmPsGEF activates Rac1; the active GTP·Rac1 is involved in the axon development in MB neurons via a Pak-independent signaling pathway.

### DmPsGEF functions as a GEF for Rac in cultured cells

**Figure 8:**
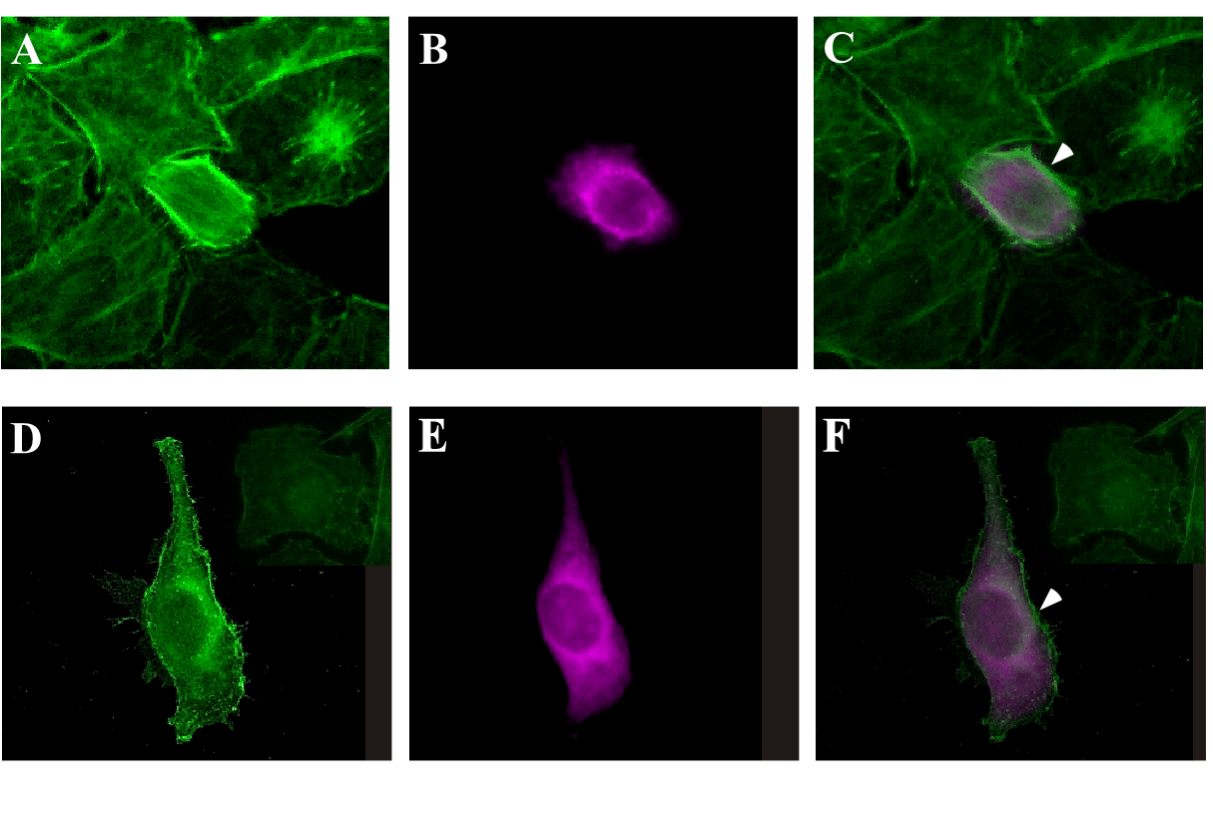
**PsGEF functions as a GEF for Rac in cultured cells**. HeLa cells transiently transfected with DmPsGEF RhoGEF domain expression constructs were stained with fluorescein isothiocyanate-phalloidin to detect F-actin **(A and D)**, and anti-myc antibodies were used to detect the RhoGEF domain of DmPsGEF **(B and E)**. The images A and B are merged to form image **(C)**, and the images D and E are merged to form image **(F)**. Actin polymerization in membranes, which results in membrane ruffling, is specifically observed in cells that express the RhoGEF domain of DmPsGEF (arrowheads in C and F).

To test whether PsGEF is a GEF for Rac, the RhoGEF domain of DmPsGEF was expressed in HeLa cells, and the F-actin of these cells was visualized by using fluorescein isothiocyanate (FITC)-phalloidin. The RhoGEF activity of PsGEF is likely to be affected by the intracellular Ca^2+ ^level as well as its interaction with other proteins through the C2 and PDZ domains. Thus, only the RhoGEF domain of DmPsGEF was expressed in the HeLa cells. Actin polymerization in membranes resulting in membrane ruffling is specifically observed in the cells expressing the RhoGEF domain of DmPsGEF (Figures 8C and 8F). These phenotypes are similar to those obtained with the expression of active forms of Rac but not Cdc42 or Rho [27]. The active forms of Cdc42 and Rho are known to induce filopodia and stress fibers, respectively [28]. These results therefore suggest that the RhoGEF domain of DmPsGEF activates Rac but not Cdc42 or Rho in cultured cells. This finding is consistent with the genetic interaction of *DmPsGEF *with *Rac1 *but not *Cdc42 *or *Rho1*, as described earlier.

## Discussion

### Genes specifically necessary for the development and/or functions of the insect nervous system

We are interested in discovering the specific molecular features underlying the functions of insect nervous systems. There are several properties specific to the insect nervous system. One of the characteristics of the holometabolous insect brain is the development of the brain from the larval to the adult stages during metamorphosis. Although the overall brain organization is preserved during metamorphosis, dramatic remodeling of neuronal circuits occurs. Some embryonic and early larval neurons die, and others undergo axon pruning and regrowth. Furthermore, many neurons are born during metamorphosis [29]. The genes involved in neuronal remodeling and metamorphosis might be specific to the insect genomes. With regard to the structural characteristics of the insect brain, MBs are specific neuropil structures found in many marine annelids and almost all arthropod groups, except crustaceans [11]. MBs are lobed neuropils that comprise long and approximately parallel axons originating from clusters of minute basophilic cells located dorsally in the most anterior neuromere of the CNS. MBs are higher olfactory and multisensory centers of the insect brain and are implicated in odor discrimination and in olfactory and other forms of associative learning [12]. It is not known how MBs evolved in different taxa. There may be some common genes among annelids and insects, and they may have a role in the evolution of MBs. *PsGEF *may be one of these genes.

### Origin and evolution of PsGEF

We first identified *PsGEF *as an insect-specific gene, which is highly expressed in the *Drosophila *CNS. It is present in the sequenced genomes of insects but not vertebrates. A search for *PsGEF *orthologs in other sequenced genomes revealed that *PsGEF *is present in the parasitic wasp, the human body louse, and, surprisingly, the limpet *Lottia*. Due to the phylogenetic distance between insects and limpets, it is unlikely that *PsGEF *independently evolved in these taxa. In fact, a domain-by-domain phylogenetic analysis of RhoGEFs from different species demonstrates that the C2, PDZ, and RhoGEF domains of LgPsGEF cluster with those of insect PsGEFs, and do not branch with those of different families (data not shown). Thus, it is unlikely that PsGEFs have been independently generated by exon shuffling (or exon capture) in the lophotrochozoan and insect lineages. The exon-intron structures of five *PsGEFs *from different species also support this conclusion since the positions as well as phases of some introns are conserved in the C2, PDZ, and RhoGEF domain-coding regions (Figure 2). This may suggest that the last common ancestor of protostomes gained the intron-rich ancestor of *PsGEF*, and some of these ancient introns have been lost, and some new introns have been gained in each species, as demonstrated with, for example, nuclear OXPHOS genes [30].

Apparently, intron loss has been most extensive in *DmPsGEF. PsGEF *was absent in Urbilateria, and then specifically acquired by the common ancestor of all protostomes but not deuterostomes or cnidarians. How was the ancient *PsGEF *originated? Since vertebrates possess RGS3 with C2 and PDZ domains similar to the short form of DmPsGEF, this gene was present in Urbilateria. It then acquired the exons encoding the RhoGEF domain by exon shuffling or exon capture from the different families to generate *PsGEF *[31]. Intriguingly, *PsGEF *is absent in nematodes, suggesting that it has been secondarily lost from some ecdysozoan species. Since it has been demonstrated that the evolutionary rates of insects and nematodes are fast and comparable [2,3,32], it is difficult to imagine that nematodes evolved an alternative pathway or paralogs of *PsGEF *to compensate for the absence of PsGEF and insects failed to evolve such systems. A more plausible explanation is that *PsGEF *has been selectively retained in the insect and limpet genomes because it continues to play an important role in these animals but not in nematodes.

An analysis of the *Daphnia *genome revealed that the *Daphnia *PsGEF contains only the C2 and PDZ domains, and it corresponds to the short form of DmPsGEF. The exons encoding RhoGEF domains are apparently missing in the *Daphnia *genome (Figure 3). This demonstrates that the exons encoding the RhoGEF domain were specifically deleted in crustaceans after split from a common ancestor of crustaceans and insects [33,34]. These results suggest that *Daphnia *PsGEF cannot function as a RhoGEF, which is critical for MB axon development (Figure 7 and see below). Thus, PsGEF containing the C2, PDZ, and RhoGEF domains appears to be present only in the limpet *Lottia *and insects but not in crustaceans. Although it is not known whether MB-like structures are present in the limpet brain, this distribution pattern among metazoans coincides with the presence of MBs in the brains of certain platyhelminthes, marine annelids, and insects but not crustaceans [35]. These results suggest that PsGEF may be associated with the evolution of MB-like brain structures and is, in fact, necessary for MB axon development in *Drosophila *(Figure 6).

### Molecular functions of PsGEF

Alternative splicing of *DmPsGEF *determines the presence or absence of the RhoGEF domain in the protein (Figures 1A and 1B). The activation of Rac by PsGEF is therefore directly regulated by alternative splicing. What are the functions of the short version of PsGEF with only the C2 and PDZ domains, which is also present in *Daphnia*? If both short and long PsGEFs are present in a single neuron, the short protein may exert dominant negative effects on the long protein. The short and long proteins may compete for binding with Ca^2+ ^and certain proteins through the C2 and PDZ domains, respectively. This, in turn, regulates the RhoGEF activity of the long PsGEF. If their expression is mutually exclusive, they may have independent functions in different neurons. Since it is difficult to distinguish the expression patterns of short and long *DmPsGEF *mRNAs in *Drosophila*, an examination of the functions of short PsGEF in *Daphnia *may provide an answer to the abovementioned question. Although alternative splicing of *PsGEF *occurs in the fruit fly and honey bee (data not shown), it remains to be established whether the same alternative splicing occurs in other insects and limpets.

Loss of *DmPsGEF *results in thinner alpha lobes than those of wild-type (Figures 6 and 7). Furthermore, short and multiple alpha lobes derived from late-born *201Y-Gal4*-expressing alpha/beta neurons were observed in MBs of *dmPsGEF*^Δ*21 *^flies, as shown in Figures 6C to 6E. These results suggest that the alpha/beta neurons bifurcate their axons at more random positions in *dmPsGEF*^Δ*21 *^flies. Further, the *dmPsGEF*^Δ*21 *^alpha/beta neurons may not respond well to the signals provided by guidance neurons (the pre-existing alpha/beta neurons). Moreover, some alpha/beta neurons fail to extend their alpha-axonal branches (Figures 6F to 6H). Meanwhile, gamma neurons appear to be normal. Alpha'/beta' neurons are generally born during the late third instar, and their axons (alpha'/beta' lobes) remain intact during metamorphosis [25]. These lobes may serve as the guiding axons for the bifurcation of the axons of alpha/beta neurons. We therefore examined the alpha'/beta' lobes in *dmPsGEF*^Δ*21 *^flies by immunostaining with anti-Trio antibody [24]. Their morphology was found to be normal, thus suggesting that *DmPsGEF *is specifically necessary for the axonal development of alpha/beta neurons in MBs.

To identify Rho family members activated by PsGEF, we first tested the genetic interaction of *DmPsGEF *with five *Rho *family members. The results indicated that *DmPsGEF *genetically interacts with *Rac1 *but not other family members (Figure 7). Consistent with this observation, the ectopic expression of the RhoGEF domain of DmPsGEF induces actin polymerization in the membrane of HeLa cells (Figure 8). These results thus suggest that PsGEF activates Rac but not Cdc42 or Rho *in vivo*. Intriguingly, *DmPsGEF *does not genetically interact with *Pak*, one of the downstream effectors of Rac (Figure 7I). Trio was shown to activate Rac and promote LIM kinase activity via Pak to induce axon growth inhibition [36]. Thus, DmPsGEF induces Rac activation and may stimulate axon growth via a Pak-independent pathway along with Still life (Sif) [36,37]. Three GEFs for Rac (Trio, Sif, and DmPsGEF) appear to function for the morphogenesis of MBs in *Drosophila*. Since each GEF protein has specific functional domains (for example, Spectrin repeats and SH3 domain in Trio, PH and PDZ domains in Sif) in addition to the RhoGEF domain, the mechanism to activate individual GEFs should be different. Specifically, the presence of the C2 domain in PsGEF suggests that the increase of intracellular Ca^2+ ^level is essential for the localization of PsGEF at the plasma membrane where Rac proteins are anchored. The function of DmPsGEF is therefore dependent on the local increase of cytosolic Ca^2+ ^level which occurs during the axonal development of alpha/beta neurons in MBs.

*DmPsGEF *is expressed not only in the MBs but also in the antennal and optic lobes in adult *Drosophila *brain. We analyzed the axonal projection patterns of several olfactory neurons into the antennal lobes in *dmPsGEF*^Δ*21 *^flies; however, no significant difference was observed relative to the patterns observed in the wild-type (data not shown). The glomerular structures of the antennal lobes visualized by immunostaining with anti-nc82 antibody were found to be normal (data not shown). Although we did not observe gross morphological defects in the antennal and optic lobes in *dmPsGEF*^Δ*21 *^flies, it is possible that there were subtle defects in these brain regions. No other proteins with C2 and PDZ domains are present besides DmPsGEF in *Drosophila*. However, other GEFs for Rac (Trio and Sif) could be partially redundant with DmPsGEF. This may explain the lack of phenotypes associated with the antennal lobes in *dmPsGEF*^Δ*21 *^flies. We are also testing the olfactory and visual behaviors of adult *dmPsGEF*^Δ*21 *^flies at present.

### Association of PsGEF with structural and/or functional features common between insect and lophotrochozoan nervous systems

**Figure 9:**
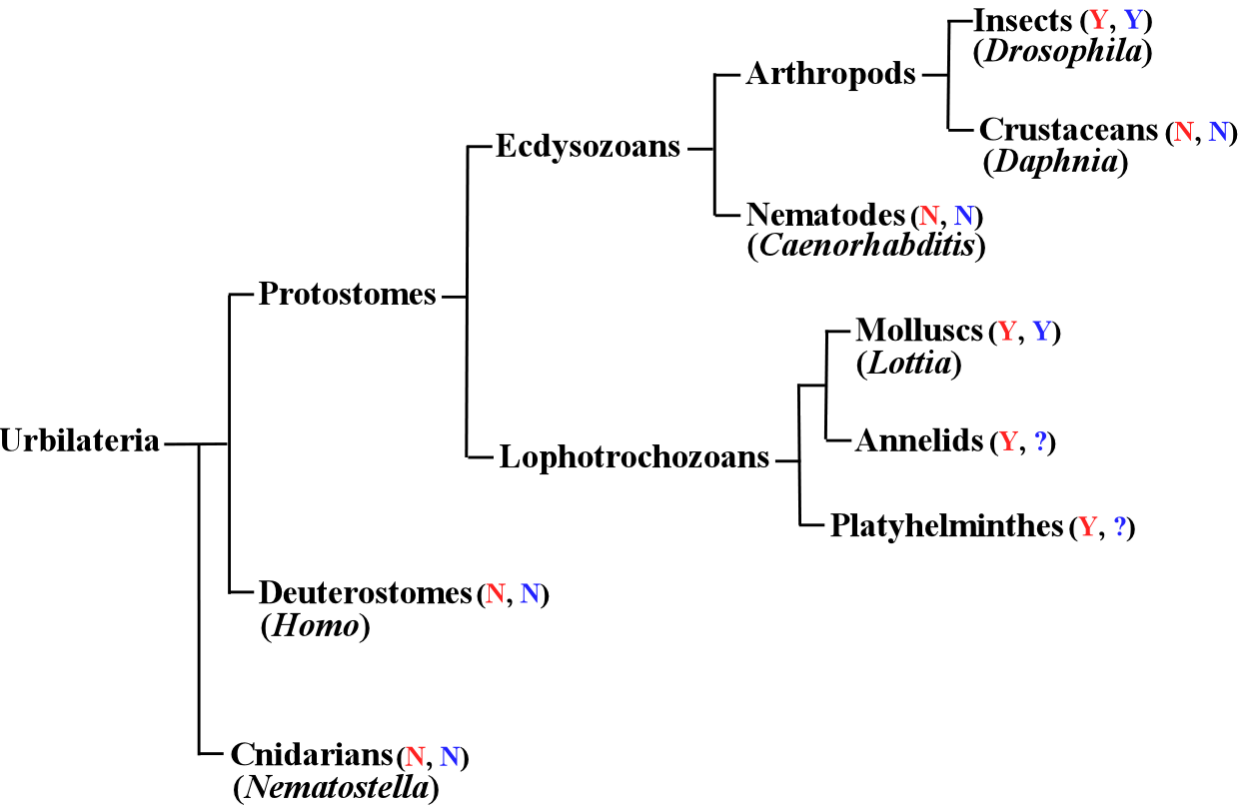
**The phylogeny of animals described in the text**. The presence and absence of mushroom body-like structures are indicated with Y and N in red, respectively. The presence and absence of PsGEF with C2, PDZ, and RhoGEF domains are shown with Y and N in blue, respectively. The status is not known in annelids and platyhelminthes, and thus indicated with ?. Representative animal species are also shown in parentheses. Not all animal phyla are indicated.

The specific acquisition of *PsGEF *by the last common ancestor of protostomes followed by the retention or loss in specific animal species during evolution demonstrates that there are some structural and/or functional features common between insect and lophotrochozoan nervous systems, which are absent in all deuterostomes and cnidarians. We would like to propose that *PsGEF *may be associated with the presence of MBs, specific brain structures in insects, annelids, and platyhelminthes [35]. The fact that PsGEF containing the C2, PDZ, and RhoGEF domains is specifically retained in animals having MBs (for example, insects but not crustaceans in arthropods) in addition to functioning as a GEF for Rac, which is essential for the correct axonal development of MBs in *Drosophila*, suggests that the last common ancestor of protostomes might have possessed an ancient MB-like structure in the nervous system, and the MBs found in present animals may have evolved into specific neuropil structures in insect and lophotrochozoan brains supported in part by the retention of *PsGEF *in their genomes during evolution. Nematodes and crustaceans lost the full length *PsGEF*, and MB-like structures have disappeared as a result (Figure 9). However, the functions of PsGEF do not appear to be limited to MBs because it is also expressed in the optic and antennal lobes of adult *Drosophila *brain. Although MB-like structures are found in mollusks such as *Achatina *[38] and *Octopus *[39], it is not known whether limpets also have MB-like structures in their brains. It is thus possible that the conservation of *PsGEF *could be for other functional and developmental constraints.

Recent studies have demonstrated that the expression profile and roles of genes patterning the nervous system in the embryos of vertebrates and annelids are quite similar [9]. Since it is unlikely that this remarkable similarity arose from convergent evolution, they suggest that Urbilateria may have already had a quite complex CNS, which is also supported by comparative genomics [3,10]. It is therefore not surprising that the origin of MBs could be traced back to the last common ancestor of protostomes, in which ancient MB-like structures might have played a role in multimodal sensory integration and even learning and memory. To prove a single origin of MBs, it will be necessary to demonstrate that the same gene sets (for example, *ey, toy*, and *dac*) act similarly for the development of MBs in insects and lophotrochozoans. The homologs of these genes are also present in the vertebrate genomes, and they function in the early development of nervous systems. As these genes encode transcription factors, they could function as a common 'genetic code' to specify the brain region, giving rise to MBs and its vertebrate equivalents. Then, as a next level, a different set of genes such as *PsGEF *is necessary to generate the specific structures of MBs, which are present in the brains of insects and lophotrochozoans but not vertebrates. In this regard, *PsGEF *is one of genes involved in generating the diversity of metazoan nervous systems.

## Conclusion

The specific acquisition of *PsGEF *by the last common ancestor of protostomes followed by the retention or loss in specific animal species during evolution demonstrates that there are some structural and/or functional features common between insect and lophotrochozoan nervous systems (for example, MBs), which are absent in all deuterostomes and cnidarians. *PsGEF *is therefore one of genes associated with the diversity of metazoan nervous systems.

## Methods

### Screening of insect-specific genes highly expressed in the Drosophila central nervous system

The identification of insect-specific orthologous genes has been previously described [1]. *Drosophila *genes were analyzed using FlyBase [40] in order to identify those in which *GAL4 *was inserted in their putative promoter regions. These *GAL4 *enhancer trap lines were individually crossed with *UAS-mCD8::GFP*; this was then followed by examination of green fluorescence protein (GFP) expression patterns in their progenies. We identified one line, namely, *NP5114*, which exhibits strong GFP expression in the embryonic and adult *Drosophila *CNS (Figure 4). In this line, *GAL4 *was inserted at a position 5' upstream of the first exon of *DmPsGEF (CG14045) *on the X chromosome (Figure 5). Other *GAL4 *lines in which *GAL4 *was inserted close to *NP5114 *(*NP0264, NP1088, NP7169, NP3316, NP7265, NP3612*, and *NP3237*) exhibit the same expression patterns, with variable intensities.

### Identification of alternatively spliced DmPsGEF mRNAs

There is one *DmPsGEF *cDNA sequence deposited in an NCBI database (RE74757). This sequence corresponds to the short mRNA encoding DmPsGEF with the C2 and PDZ domains; this was confirmed by RT-PCR and rapid amplification of cDNA ends (3' RACE). To verify the presence of long mRNAs encoding DmPsGEF with the C2, PDZ, and RhoGEF domains, the corresponding full-length cDNA was isolated by RT-PCR and then sequenced. The obtained sequence is identical to the one predicted from the genomic sequence (*CG14045*), except that a 108-bp sequence is absent.

### Bioinformatics

The amino acid sequences of TcPsGEF (GLEAN_01044), AmPsGEF (GB16089-PA), PhcPsGEF (PHUM010260-PA), and LgPsGEF (fgenesh2_pg.C_sca_68000085) were retrieved from Baylor [41], BeeBase [42], VectorBase [43], and JGI [44], respectively. The functional domains of each protein were analyzed by NCBI CD search, and the sequence alignment of five PsGEF proteins was done by MEGA4 [45]. The genomic sequences of five *PsGEF *were also retrieved from above databases and FlyBase.

### Genetics

*DmPsGEF *deletion mutants were generated by the imprecise excision of *NP5114*. Approximately 300 *w *female revertants balanced with FM7 were individually crossed with FM7 males to establish the lines. The *B*^+ ^adult males were collected from each line, and their genomic DNA was analyzed by PCR with the following two primers, namely, 5'-CACGGGATCTGCAGTGCAGACAACTCTT-3' and 5'-CAATCGCAGCTGTCAGTTCGGGAGGTGC-3', to identify the deletion mutants. The genomic PCR yielded a 7.5-kb band from the wild-type; thus, lines yielding bands smaller than 7.5 kb were analyzed further. We identified two large deletion mutants, namely, *dmPsGEF*^Δ*55 *^and *dmPsGEF*^Δ*21*^, and their breakpoints were determined by sequencing.

In order to visualize the alpha/beta lobes of MBs by GFP, *y, w, dmPsGEF*^Δ*21*^; *201Y-GAL4 *females were crossed with *UAS-mCD8::GFP *males, and the resulting males were examined. To analyze the genetic interaction of *DmPsGEF *with *Rac1*, *Rac2*, *Mtl*, *Rho1*, and *Pak*, the *y*, *w*, *dmPsGEF*^Δ*21*^females were crossed with *y*, *w; rac1*^*J11*^/*CyO*, *y*, *w*; *rac2*^Δ^, *y*, *w*; *mtl*^Δ^/*TM3*, *y*, *w*; *rho1*^*E3.10*^/*CyO*, and *y*, *w*; *pak*^6^*/TM3 *males. The resulting males were then examined. To analyze the interaction of *DmPsGEF *with *Cdc42*, we crossed *FM7/y*, *w*, *dmPsGEF*^Δ*21*^, *cdc42*^4 ^females with *y*, *w*, *dmPsGEF*^Δ*21 *^males, and then the resulting *B*^+ ^females were analyzed. More than 30 animals were analyzed for each case.

Single-cell MARCM clones (alpha/beta neurons) were generated by heat-shocking late-stage pupae of *y*, *w*, *dmPsGEF*^Δ*21*^, *FRT19A/w*, *hs-FLP*, *tub-GAL80*, *FRT19A*; *UAS-mCD8::GFP/+; OK107/+ *at 37°C for 45 min [25]. As the wild-type control, *y, w, FRT19A *chromosomes were used.

### Immunohistochemistry

The embryos were immunostained with rabbit anti-GFP antibodies (1,000-fold dilution) and horseradish peroxidase-conjugated anti-rabbit IgG antibodies (300-fold dilution) by using 3,3'-diaminobenzidine and nickel chloride as previously described [46]. The adult brains were dissected and fixed with 4% paraformaldehyde/PBS on ice for 3 h, permeabilized with phosphate-buffered saline (PBS) containing 0.5% TX-100 for 5 min, and then blocked with PBS containing 5% normal goat serum for 30 min. They were then immunostained with 1D4 (4-fold dilution), rabbit anti-GFP antibody (1,000-fold dilution), Rhodamine anti-mouse IgG (300-fold dilution), and FITC anti-rabbit IgG (300-fold dilution).

### Reverse transcriptase-polymerase chain reaction

Total RNA was isolated from *NP5114*,*dmPsGEF*^Δ*55*^, and *dmPsGEF*^Δ*21 *^embryos. Thereafter, cDNAs were synthesized by using a reverse transcriptase (ReverTra Ace, TOYOBO). PCR was carried out using the following primers: 5'-ATGACACGGATGCATCGCCACTCCAGTT-3' and 5'-TTAGACGAAGACACCTTTGCCTACCTCC-3' (for *DmPsGEF *short mRNA), 5'-ACCTTCAGCAAGGAGTCGATTGTGCCTG-3' and 5'-CTGCAGTTCGTTGATAACCGTGCTAAAG-3' (for *DmPsGEF *long mRNA), and 5'-TGAGCATGAGCGCCACCTCGGATATCTA-3' and 5'-TGAGACTGGCGGATCTAGATGACGTAGT-3' (for *CG14047 *mRNA). The resulting PCR products were sequenced to verify their identities.

Total RNA was isolated from frozen samples of *Daphnia pulex*, and the cDNAs were then synthesized as mentioned above. PCR was carried out using the following primers: 5'-ATCGGCTCGCTACCTGAAATCCAACAGC-3' and 5'-GTGACGCTTCCGCCTCCTGACGGTTTCT-3' (for *SNAP_00018439 *mRNA), 5'-ATGGCGGCCTCGTCTTCGGACCTTCAGG-3' and 5'-TCAAGTATCCTCGCAGCGTTCACCGAGT-3' (for *SNAP_00018441 *mRNA), and 5'-GGTATCATGTCCGTGCAGCTGCACAAGT-3' and 5'-CATCCATTTGACGGCGGATAAGGTCGAC-3' (for *SNAP_00018442 *mRNA). The resulting PCR products were sequenced to verify their identities.

### Ectopic expression of RhoGEF domain of DmPsGEF in HeLa cells

A DNA fragment encoding the RhoGEF domain of DmPsGEF was PCR-amplified using *DmPsGEF *long cDNA as a template and the following primers: 5'-TTTTTGCGGCCGCATGAGCCGGCCGGCTACCGCATGCTCGG-3' and 5'-TTTTTTAAGCTTACCAATATCACTCAGCGGCACCAGGGTG-3'. The resulting PCR product was digested with *Not*I and *Hind*III and inserted at the sites of the pCMVTag5A vector (Stratagene) that were treated with the same restriction enzymes. The resulting expression construct was introduced into HeLa cells with Effectene transfection reagent (Qiagen). Two days after transfection, the cells were fixed with 4% paraformaldehyde/PBS at room temperature for 15 min; this was followed by permeabilization and blocking as done before. The transfected cells were detected by using rabbit anti-myc antibody (1,000-fold dilution) and Rhodamine anti-rabbit IgG (300-fold dilution), and the F-actin was stained using FITC-phalloidin.

## Authors' contributions

NH and KK performed all experiments described here. TK designed the experiments and wrote the manuscript. All authors have read and approved the final manuscript.

## Supplementary Material

Additional file 1 **Supplementary material**. The full length amino acid sequences of short and long DmPsGEF, TcPsGEF, PhcPsGEF, AmPsGEF, and LgPsGEF proteins are shown by a FASTA format. Click here for file

Additional file 2 **Figure S1**. Alignment of amino acid sequences containing the C2, PDZ, and RhoGEF domains of five PsGEF proteins. The amino acid sequences containing the C2, PDZ, and RhoGEF domains of DmPsGEF (amino acid 272 to 1132), TcPsGEF (amino acid 601 to 1313), PhcPsGEF (amino acid 14 to 700), AmPsGEF (amino acid 595 to 1238), and LgPsGEF (amino acid 18 to 730) proteins are aligned by CLUSTALW program. The amino acid sequences of C2, PDZ, and RhoGEF domains are highlighted by red, green, and blue, respectively. Identical amino acids are indicated by asterisks, and the conserved amino acids are shown by either dots or colons. Only C2, PDZ, and RhoGEF domains show the significant similarity between five PsGEF proteins. Click here for file
