# Supplementary material for: Specific retention of the protostome-specific PsGEF may parallel with the evolution of mushroom bodies in insect and lophotrochozoan brains
Source: BMC Biol. 2009 May 7;7:21. doi: 10.1186/1741-7007-7-21 (PMC2684095; doi:10.1186/1741-7007-7-21)
Supplement: Additional file 1 — Supplementary material. The full length amino acid sequences of short and long DmPsGEF, TcPsGEF, PhcPsGEF, AmPsGEF, and LgPsGEF proteins are shown by a FASTA format. [file 1741-7007-7-21-S1.pdf]

>Short DmPsGEF
MPTMTRMHRHSSSSAVVEESRGRRRGVGVPGVGDANKENFGVHFMSSPFGNASLIALQDLSNVHGKSPQR
RSFSEGSGPRQATPQLAALRCLPRTTGGAVALEDSQLSSSRMGDTTLDRMLDAIIESARKEVRCTKTLPG
AGATTSTTILADNAEWSETSVHEMEVRTPTHLKRQRVVRRKNPHKTTTINQTQSHQAKKLEPLQLVPSTK
RCLSFSSSSASSDLDEDEQQVAKRSSLASPTTTPPSHCTTTTSSISSSSSSNGGADMEASQRGSIDVSIA
FDAKEQQLNVHVIRCRDLQRSHGSGNGSINAYVKVALSGGAQPPGYGGHSSGGSMSSGYQRTAVHRHSGR
PYFDQRFNFQISSGEETAGQYLQLAVWHRDRHLKRSEFLGCSTFPLNELVHPDSGVSAGSYKLHAQACPP
PTSRHSQPKANAGQDQKQETEAAKDQDKQQDDSPAEMAAAVTVTPQKPVATGSGSGSNSAAMALNDEVIS
ISIDSMGKEDPLLPQQPQQPMKLSKKALHQRDADENLFLRFLELDPPADGNANSTTTQAQATGSQSSASK
ANESNANHLNNGTSGGRRQSTMPNSGGSSVGGAVRQQQGRTPFTMTKRLTRTEERGFGFSIVWTHPPRVE
KIEAGLSADRCGILPGDYVIFVDKHNVVTMPEADVLNLIRSQGSSLTLEIFRRSGAGATTITSTDLGQNN
VHISTRLGAAVGLGSEEHTLATTATGTTTVMSLQRTTSTRIQPLANSMSRPATACSGTTSSIEAAKRRLH
LPQVTFSKEVGKGVFV

>Long DmPsGEF
MPTMTRMHRHSSSSAVVEESRGRRRGVGVPGVGDANKENFGVHFMSSPFGNASLIALQDLSNVHGKSPQR
RSFSEGSGPRQATPQLAALRCLPRTTGGAVALEDSQLSSSRMGDTTLDRMLDAIIESARKEVRCTKTLPG
AGATTSTTILADNAEWSETSVHEMEVRTPTHLKRQRVVRRKNPHKTTTINQTQSHQAKKLEPLQLVPSTK
RCLSFSSSSASSDLDEDEQQVAKRSSLASPTTTPPSHCTTTTSSISSSSSSNGGADMEASQRGSIDVSIA
FDAKEQQLNVHVIRCRDLQRSHGSGNGSINAYVKVALSGGAQPPGYGGHSSGGSMSSGYQRTAVHRHSGR
PYFDQRFNFQISSGEETAGQYLQLAVWHRDRHLKRSEFLGCSTFPLNELVHPDSGVSAGSYKLHAQACPP
PTSRHSQPKANAGQDQKQETEAAKDQDKQQDDSPAEMAAAVTVTPQKPVATGSGSGSNSAAMALNDEVIS
ISIDSMGKEDPLLPQQPQQPMKLSKKALHQRDADENLFLRFLELDPPADGNANSTTTQAQATGSQSSASK
ANESNANHLNNGTSGGRRQSTMPNSGGSSVGGAVRQQQGRTPFTMTKRLTRTEERGFGFSIVWTHPPRVE
KIEAGLSADRCGILPGDYVIFVDKHNVVTMPEADVLNLIRSQGSSLTLEIFRRSGAGATTITSTDLGQNN
VHISTRLGAAVGLGSEEHTLATTATGTTTVMSLQRTTSTRIQPLANSMSRPATACSGTTSSIEAAKRRLH
LPQVTFSKESIVPVTDNRRRFLLQLISREQNFTAALHFGVDRFVQPLGERKDLISPNDHRTLFQNIDELL
RIAEDILEQLCSSDQQDQEPQMNFASRVYLSKTTAICAAYKKYCNGIKRADCVLVNKSRQTGSEFIAFIT
EPAVPRKRPDLTMFIHRPLQHFREILKLMQLLAGNCHVDTEEHKNFSTVINELQAAYREITVSSGLMEPL
GEGRPLLTLQDLESRMVFTKCKPFTLAVQGRQWIFGGDLSRVEGRSVKPYWTLLFSDIIVFAKVSRDRVL
FITEEPIPIANVVDSCFHMRKKTTEFRLTVDPNGRLAESPTGYCAPDLTRTPKRGARRKSLILRAPSLEL
KAVWQNLLQRQIFLVNAALGSTPLSSPLDSPDVLNTLVPLSDIGLTTASMGSMKLPSLDSIHLKQQQKQQ
VRLFRSKRLDSGDSYENLQRLASAVRHHCASTTTSSATQTQSHPQPPLCTTSLPSRTNSPARNQRHPNGQ
GHVGGSVPNGVGGVSFGGCNTNSSCLSSSASLTQTQTQVHTQTLNHSQTHSQTQTSVLSLSSCKCLTVIP
EVTSEPPVNQHASQKLLEFSLSSVGRSFIDESGGVVGGQVSLTTPETPTPNISPTTSQPCNSDAFSNDFV
ATSTTGKGPNIPLAEFGGSWDLLELDLQLHEVNLDPSYDTDVEECIFLGDEEDREGTEGHGHDEDSDEDD
SVVVDDPFGMLPTRPTPPDSLDL

>TcPsGEF
MRIRKYVGLLLQAKKEKFLIIPEPAGSVVKEQKSRAPECILEHGQVEQIVKNRANVEETFLPFQTRTMLK
WTVMARREADKSDALRRPTADASTSETTPKSNKTKRRHSSGHRRSGGRRSSLGLEMDKENQFTSTPIKSF
EDHHQFDALRDVSNLTPNRRVLSAKKCRRSAPSSHRKHKKKKLCLNPSHRKEFGEGKNYFRPFETVDSH

IEGVLVDKDLSGELRVKRRIQDTYAPTLPTFTTEYSPCFMRDAHCLYNFTPAKATPLGKYLALDQDTPHP
SKKPKIDHVSDFLHQINFITSPEDDKLTCPTMESKSTLTLREESKVSPLVKKLVDLRFSQLSCDKDTCKN
NSSLINDLSLDQIVDAILNSSAESAANKENESNETHEENLINTENERHVATSFDRCSSDSGFKSSTTEYS
HQLEGNFQCKCKTSTPNAALCDKTIININETFNERCVDVINPRKRPSSTPNDDNNAKRAYLDKSEEDNVY
CTLKRQRCIRRRRTDDEKKMSAKKSRKVDEPIIGDESFDSNISGKIAEDNSTPLNETFTLLTPVDNSRRF
RRCLLFESPTSLSESASTTNSSLRDVRGTMDLNIRCENDELYANIIRCKDLYRPNGKQINAYVKVALSDR
LGDNRRKRNGILQRTAVQADSSKPFFNHTFKFPLQKESLQKRLHIEVWHRDRSSRTSEFLGCMSFDVRHV
YTKDISGSYRLLPQSSGRCQNIPISVEPLDREAEEDCQPIMCESQSSVDEIMSLDGLDNDLKKATNKAIL
SEQQKYADENLFLRYLELDPTEGPEAIPAAMQRKATGNKNGRTPFTQTKKLTRAPKSGFGFSVVWTHPPR
IERVEKGLPAEKAGILPGDYIIFVDKHNVVMMPEIDILNLIRSYGSQLTLEIFRRNAAKNGSVPSVRRLN
STGTCSTTGLPISSNLVQRRPSTVCSTNTASVDYNRRKLHLPQVTFSAEKASNNQEENRTKAMYQLIHKE
QQYATGLQFAVTRFVSALAERRDLITPSEHKILFQNSEEILRVTEDILDNLVHEEGVHNLIRTYHAKLNE
ITAAYRRYCSGIKKADCVLANKTKNSNSEFVRFLHTPSIPRRRPDITAFIHKPLEHYREVLKAFTVIQSH
TKPNHEDYPVINQLVHDLQHTYREITVEAGLMEPMGEGRPLLSVQDLENRLVFTKCKPFVLNKPGRQWIF
GGDLGRVEGRNVRQYWTLLFSDLLLFAKVSRDRVLFIIEDPLPLAHITDMFFNVRKKVDSGLLDTEFRIT
VTPGGRLASSPTVHCGPDLSRTPKKNTGKRTVILRAPTTELKAVWQNLLQRQM

>PhcPsGEF
MHVHERNSRGSVRKVALVPSAERTFFRTPVHRESSCPNFNETFSFNFHSEDLNKHILVSVWHRDRDNRKS
EFLGCVSLAVKNAIKKEISGWFRLVAQSGGSKSSSNQQRPIQSNCGLSTTGSPSMMAHRSGNQKFDELIP
FDDEPRSSPPDKHNNKYSIKKKLNDDSSFLRHLELEPIDGEGGRVPLPEAIAKGGRTPFTTTKQLTKQPG
GGFGFSIAWVQPPRVERVEAGQPADRAGIRPGDFVIFVDRFNVVTMSEEEILDIIKSCGNELTLEIYRKS
NANGCLVEPPIALPSTMQTSVEYNNSSRWSGTCYAPTGTTNTTTTTTTSTSFDFAKRRLHLPQVAFTNEV
STLNPEESRKKAIYQLLSKEQNYATNLQFGINRFSVPLLERKEFINQKQHQILFQNIEEILRLTEDTLEQ
AINEEVDLTGDSIGRLYFKRIQVLKPAYRRYCSGIKRADCVLAEKFKNNEFNRFCAEPPVPRKRPDLTTF
LHKPLEHYRELLKHLQIIFNLTKSPDTDHAALSRVVNDMQSSFREVTGGLMEPEVEGRPLLSVQDLESRL
VFTRCKPFVLSIPGRQWIFGGDLSKIEGRTVHPFWALLFTDMMIFAKVSRDRVLFITEEPLSLLAVSQAF
FNIRKKANEFRLLLSNNSPGMDSPANSTTCGPDIVLSRTPKKNTKFKSIALRAPTPELKAVWQNLIQRQI
IHLNTTRGLTPSTSPLESPDPPTTISAATLDTISLKRQMRLPQVYSCSPNHVPRDDEDETSSPPPEKFYY
EPLLTPDSATTFG

>AmPsGEF
MVNIPTAGNRETPTSTFKPGDHGASTWGEWSGRGLLGSGHGTRVGVHVRVQYTDKMLKWTVSRSLSASSS
TPHGPPNLNKAPRRPFRDLSNTPPAAGTKARGIPGQNVENISARTTPVRRRRCRSHGSDMRAYFQATKPN
LRASKRQSMSQQQTEKSSESFYQGTPRVDADIGPLTFLPQGGRCPTALTLPTDPALFMRKTRVAKNFPTD
NELPQNIVNEARANLQSHVSDFFQQISMATSPEDVMEAESVPFSMARPIYPRSLNEEGGRKTSEGTRMPY
LPKLAKITRIHSRCRTPYHGKLAPSAAAKGKGHTPLLGKLSSLAIDQDLPKFENMDNTVTEVLNREQEVN
FTGKTVAEILLGDVEERKKVLGGSRGGEEENSTMKTDHTYETIAEREETAMEEEGGSCHNDSSVSSASQL
LEDPSPMSWAFASPTNDLDGEFTLKRQRGIRRKRHRKDTDKPLDGKRAKRRSTSRSPSAKTKTEAQPTLL
AEAVNRNVKKLALETDLDTTLGDEKFEPKALFCGLEGRGREEGRKTNTWDLDSPKSTLTDDFHSSKSYLN
SVTNTPETPLVATVRRCLKFSPETAEPPRPNCHGSIEIEYSVVADQIHVRVIRCKDLRRAYEGPVHAYVK
ASLKNTNGEGVVKRTAVHRATPNPVFHETLILPCPVNNNHCSSKPTSLDIAVWHRDRRARRSELLGCMTL

PLPLSQDKEATWHPLEAGTGRNTSTPYVGLPQDCGISPPLSKDGESDNNNSGGEDLTYLRHLELEPIDPL
TGLPLHPGFTAKGGRTPCTVTRRLIRQTANQIPWGFSLSWGRPPRVERVDPGSPAERSGLRPGDHVVFVD
MTNVVTRSREEILGLIQAATNQLILEVYRKGNVHSSMHRPNSMNVSLQQSIGGGHHAVAFNAEVGTGVLV
FPNLAPDAPPEEELSVREARYWGILKSGHTRFMAPLAERRDVLSAADYLILFQNLDELLKISEEIRDEGG
GVESYLCRVPRITAAYRRYLSGLQRACCLLVALRRNTAFAKLVCEPAVPHKRRPDLTGVLLLPLEHYREM
TRLLSLASPRNCQQARDLAQGYREATANAGVMEPPRDTGRPLLSLQEVESRLVFARCKPFTLAMPGRQWL
FGGALAKVEGRARLQPSWALLLTDLLVFARVSRDRVLFVTEEPLRLSNIAEACFTVRKRPTEFRLQIAIN
PAGNSENGHVEVSNSGGCSPHSRPRRRVMVLRAPTPELKAVWHNLLQRQM

>LgPsGEF
MVIDLSESTRKKYTTSGQLKVSAYMNFGLLTVHVVQGRNFSSSWKPLCDSYIKMSLVPDESKRTRCKTAS
ILNTNNPLFDDKFSFEILEEDHNKRLLISVWHKDQKSGLSEFLGCMSFGVKHLMNPKKEVNGWYYLLTEE
IGRKKHLQITKTQKPALKTNTQSNIPQINKDVKGSEPTTVMMYRGKNGFGFSVVESFPVKVGRVDGASPA
EEAGLIQGDTIVKVNGQNVSRSTAVSVAKLVKRSSNKLVLDVQRITENSTNNTYEKPIRHNNSPEPIYES
INSYASYPSQDLDSTDGDISREMIRDSEISVDTDNAFLDDPEEFMPTSTPLPLLCNGHRTVTSVNEQRKQ
EAVHRLLSLELDFIDFMHAGVQRYSRPLRHCILAAQQHSSLFQNVEKLVTISEYHVKQMQDNSPSFYSDA
DDTQSSDGHFFHVMGLIYQSKLHMLCQAYEIYANGLSNANAVLSDLKRNPDFVRFVKEPVLTQGVPSISA
FIYRPIQHLKELQQCLQDIFNNTSTDSEDFITLKNVVEGLQESVNNMTNCSTRVHSLNSLSSKRSKGSAG
SLGSSGSSSGSGSMKYGSSSSGSSSSNKVPNSCSMQTVRSVDAEVMKIQDRLVFDTNIPVFQLCQEERHL
IYRGDTFKWEGQQWVKIHMLLFSDVLLQVEKDRSGFMKVIEEPLFLREICGVEANRKHATEFLLHTCPRS
PCTGLPAPRRLVFCASTTEEKCVWKNLLEQRVHNIRGTITQYSSTSSDNSISSVIV
